# Chronic ethanol exposure increases microtubule content in PC12 cells

**DOI:** 10.1186/1471-2202-6-16

**Published:** 2005-03-11

**Authors:** Cindy K Reiter-Funk, Douglas P Dohrman

**Affiliations:** 1 Dept. of Human Anatomy and Medical Neurobiology, College of Medicine, Texas A&M University System Health Science Center, College Station, TX 77843, USA

## Abstract

**Background:**

Chronic ethanol exposure has been shown to result in changes in neuronal cyto-architecture such as aberrant sprouting and alteration of neurite outgrowth. In PC12 cells, chronic ethanol treatment produces an increase in Nerve Growth Factor (NGF)-induced neurite outgrowth that appears to require the epsilon, but not delta, isoform of Protein Kinase C (PKC). Neurites contain a core of microtubules that are formed from polymerization of free-tubulin. Therefore, it would be expected that an increase in neurite outgrowth would correlate with an increase in microtubule content. We examined the effect of chronic ethanol exposure on microtubule content in PC12 cells and the role of PKC epsilon and delta in ethanol's effect on microtubule levels.

**Results:**

Chronic ethanol exposure of wild-type and vector control PC12 cells resulted in a significant increase in microtubule content and a corresponding decrease in free tubulin. There was also a significant increase in microtubule content in PC12 cells expressing a dominate-negative inhibitor of epsilon PKC; cells which have previously been shown to have no ethanol-induced increase in neurite outgrowth. In contrast, ethanol had no effect on microtubule content in PC12 cells expressing a dominate-negative inhibitor of delta PKC.

**Conclusion:**

These results suggest that chronic ethanol exposure alters the relative ratio of free tubulin to microtubule-associated tubulin, an important component of the cytoskeleton. Further, the data from the PKC dominant-negative cell lines suggest that the effects of ethanol on microtubule content do not correlate with the effects of ethanol on neurite outgrowth. The delta isoform of PKC appears to be necessary for the ethanol-induced increase in microtubule content. These studies demonstrate an effect of chronic ethanol exposure which may contribute to previously documented alterations of neuronal cyto-architecture.

## Background

Chronic ethanol exposure has been shown to cause damage to the adult and developing nervous system [1,2]. For example, *in vivo *chronic ethanol has been shown to cause aberrant sprouting of hippocampal neurites in developing rats [3], increase the length of dendrites in cerebellar Purkinje neurons [4], the size of synaptic terminals of cerebellar granule cells [5], and the number of dendritic spines on hippocampal dentate granule neurons in adult rats [6]. Furthermore, *in vitro *ethanol enhances neurite outgrowth in cultured rat cerebellar neurons [7]. Contrary to the enhancement of neurite outgrowth, other studies have shown that chronic ethanol exposure inhibits the growth of dendrites in CA1 hippocampal neurons and cerebellar Purkinje cells *in vivo *and inhibits chick spinal cord neurite formation *in vitro *[8,9]. However, the mechanisms underlying this alteration of dendrite formation induced by ethanol exposure remain unknown.

PC12 cells have been used as a cell culture model system to study the underlying mechanisms of ethanol's alteration of neurite outgrowth [10,11]. PC12 cells are a rat chromaffin cell line that differentiate into neuronal-like cells in the presence of Nerve Growth Factor (NGF) [12]. Using these cells, chronic ethanol has been shown to enhance NGF-induced neurite outgrowth [10,11]. Thus, PC12 cells have proven to be a valuable system for studying the mechanisms underlying ethanol-induced enhancement of neurite outgrowth.

Nerve growth factor-induced neurite outgrowth in PC12 cells involves an induction of microtubule assembly [13,14]. Microtubules are formed from α and β tubulin proteins, which form head to tail protofilaments [15]. Studies have shown that Protein Kinase C (PKC) activation enhances the polymerization of tubulin to form microtubules [16-19]. Schultz et al. [20] have also demonstrated that microtubules containing phosphorylated tubulin are more stable than those containing unphosphorylated tubulin, although it remains unclear whether tubulin phosphorylation is the cause or the result of microtubule stabilization. PKC also modulates the activity of several microtubule associated proteins, including those involved in microtubule polymerization and vesicle transport [21-24].

Specific isoforms of PKC have also been implicated in mediating NGF-induced neurite outgrowth. Using both antisense oligonucleotides and specific inhibitors of PKC delta, Corbit et al. [25] have demonstrated that this isoform of PKC is required for NGF-induced neurite outgrowth. Other studies have found that in PC12 cells which over-express PKC epsilon, there is an enhancement of NGF-induced neurite outgrowth, while PC12 cells which over-express a dominant negative inhibitor of PKC epsilon show an inhibition of neurite outgrowth [26,27]. Thus, both the epsilon and delta isoforms of PKC have been implicated in modulation of NGF-induced neurite outgrowth.

It is possible that the enhancement of NGF-induced neurite outgrowth produced by chronic ethanol may be due to ethanol's known alterations of PKC signaling. Chronic ethanol has multiple effects on PKC, including altered PKC subcellular localization following chronic exposure [28,29]. Messing et al. [30] have shown that chronic ethanol exposure actually increases total cellular content of PKC delta and epsilon (membrane associated and cytosolic) in PC12 cells. However, other studies have shown that membrane-associated PKC activity is down-regulated following chronic ethanol exposure [31]. Thus, while total cellular content of PKC may increase with chronic ethanol exposure, membrane-associated PKC may be down-regulated. Interestingly, Hundle et al. [32] have shown, using PC12 cells which over-express an inhibitory fragment of either delta or epsilon PKC, that PKC epsilon is required for ethanol's enhancement of neurite outgrowth.

In this study, we examined the effect of chronic ethanol exposure on the neuronal microtubule cytoskeleton using PC12 cells as a model system. Here we show that chronic ethanol exposure increases microtubule content, while decreasing free-tubulin content. Thus, it appears that ethanol enhances microtubule polymerization in PC12 cells. We also investigated the role of microtubule polymerization in mediating ethanol's effects on neurite outgrowth using PC12 cells which over-express an inhibitory fragment of either delta or epsilon PKC. Importantly, it is the PKC epsilon isoform which is required for ethanol's enhancement of neurite outgrowth. Here, we found that the PKC delta isoform, but not the PKC epsilon isoform, is required for the enhancement of microtubule polymerization following treatment with chronic ethanol. Thus, it appears that neurite outgrowth does not correlate with enhanced microtubule polymerization in PC12 cells.

## Results

### Chronic ethanol exposure increases microtubule content in PC12 cells

**Figure 1:**
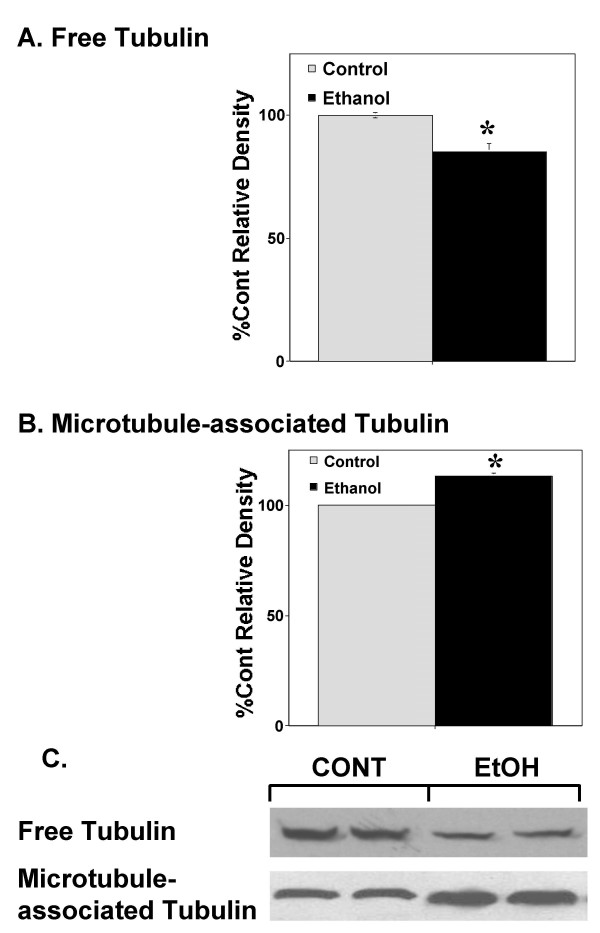
Chronic ethanol exposure increases microtubule content in PC12 cells. Chronic ethanol exposure (100 mM for 96 hours) significantly decreases free tubulin content (A) and significantly increases microtubule-associated tubulin content (B) in PC12 cells. The graphs in A and B represent the combined results of five independent replications (n = 10) of the same experiment (Three of these experiments are from wild type PC12 cells; two are from control vector transfected PC12 cells. We found no significant differences between these two cells types). Data are the mean +/- SEM. Asterisks represent significant differences from corresponding control (p < 0.001). Figure C is a representative Western Blot from one replication.

For the following studies, we used 100 mM ethanol for four days as a chronic exposure; a dose and duration used by previous researchers to demonstrate ethanol's enhancement of neurite outgrowth [10]. This is a concentration of ethanol which can easily be achieved by chronic alcoholics (0.46 g/dl). Figure 1 demonstrates that following a 96 hour exposure to 100 mM ethanol, there was approximately a 13% increase in polymerized microtubules compared to control cells (t_9 _= 5.2; p < 0.001; n = 10). Similarly, there was a significant decrease (about 15%) in free-tubulin concentration (t_9 _= 5.7; p < 0.001; n = 10) following 96 hours of ethanol exposure. There was no effect on total tubulin expression following four days of chronic ethanol exposure (t_9 _= 0.034; data not shown).

### Chronic ethanol exposure in PKC dominant-negative PC12 cells

**Figure 2:**
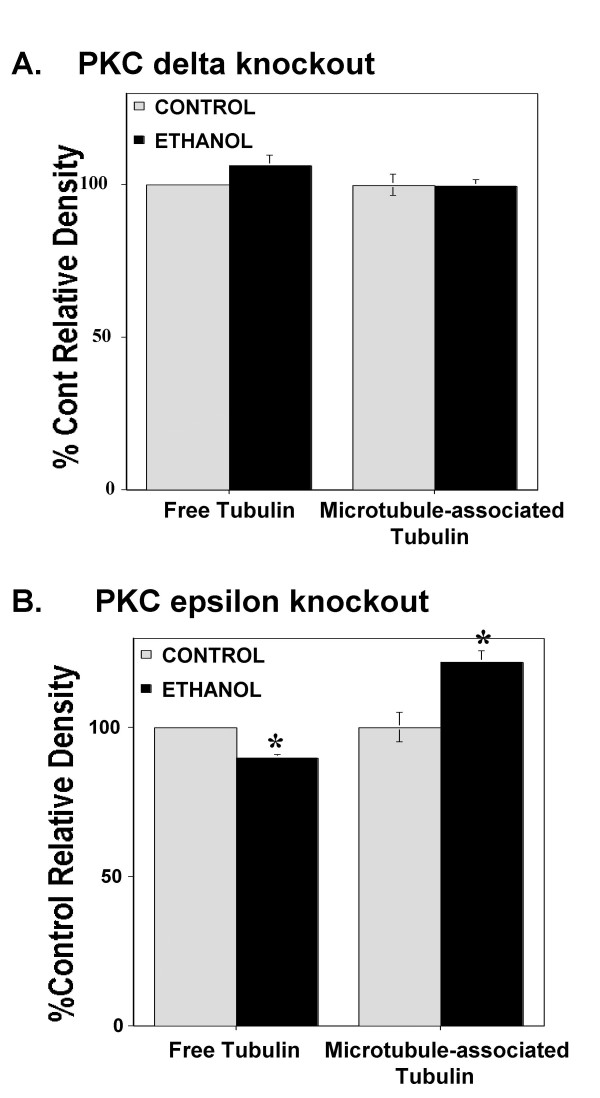
Chronic ethanol exposure in PKC dominant-negative PC12 cells. Ethanol (100 mM for 96 hours) has no effect on microtubule or tubulin content in dominant-negative delta PC12 cells (A). Ethanol significantly increased microtubule content and decreased tubulin content in dominant-negative epsilon cells (B). The graphs represent the combined data from three independent replications of the same experiment (n = 6). Data are presented as the mean +/- SEM. Asterisks represent significant differences from corresponding control (p < 0.005).

We next used PC12 cells which over-express the first variable domain of PKC epsilon or delta, which acts as an isozyme specific inhibitor of PKC epsilon or delta [32], respectively, to investigate the role of PKC in ethanol's enhancement of microtubule polymerization (Figure 2). Interestingly, in the cells which express the inhibitor of PKC epsilon (DNE cells), we found that chronic ethanol exposure significantly increased microtubule content (t_5 _= 7.15; p < 0.001; n = 6) and decreased tubulin content (t_5 _= 3.4; p < 0.01; n = 6) (similar to control PC12 cells). In the cells which express the inhibitor of PKC delta (DND cells), there was no significant effect on microtubule (t_5 _= 0.02; n = 6) or tubulin (t_5 _= 0.44; n = 6) content. Ethanol had no effect on total tubulin content in either the DNE (t_5 _= 0.2) or DND cells (t_5 _= 0.013). There was no difference between the control vector transfected PC12 cells and wild-type PC12 cells, thus these groups were combined and expressed in Figure 1. Importantly, for both the DNE and DND experiments, these experiments were replicated in two different sub-clones of transfected PC12 cells. In other words, we had two strains of dominant-negative PKC epsilon cells (DNE1 and DNE4) and two strains of dominant-negative PKC cells (DND21 and DND24). Results were similar between the two epsilon lines and between the two delta lines.

## Discussion

Cells maintain a balance between free tubulin in the cytoplasm and tubulin which is polymerized into microtubules of the cytoskeleton. In this study, we find that chronic ethanol exposure increases microtubule content while decreasing free tubulin content in PC12 cells. Ethanol appears to be enhancing polymerization of tubulin into microtubules. While there was no increase in total tubulin within the cells, there was a change in the proportion of tubulin in the polymerized (microtubule) versus non-polymerized state.

We initially hypothesized that the increase in microtubule content was due to ethanol's enhancement of neurite outgrowth. Presumably, neurite outgrowth is not due to simple stability of microtubules, but to an increase in dynamic microtubule growth. Microtubules are particularly abundant along the axons of nerve cells and multiple labs have show that chronic ethanol exposure increases NGF-induced neurite outgrowth in PC12 cells [10,11]. Therefore, since the cells that were exposed to ethanol have increased neurite outgrowth, this could be reflected as an increase in microtubles. However, it is not clear that there would necessarily be a concomitant decrease in free tubulin content or what effect of ethanol may have on microtubule stability, per se.

Our data from experiments utilizing PC12 cells which express dominant negative inhibitors of PKC do not seem to support the idea that the increased microtubule content reflects increased neurite outgrowth. Hundle et al. [32] have demonstrated the specificity of these inhibitory fragments by measuring PMA-induced translocation of PKC epsilon and delta to the particulate fraction of the cells using Western blotting with isoform specific antibodies. They show (see Figure 2 of Hundle et al. [32]) that PKC epsilon, but not PKC delta, translocation was specifically inhibited in the cells expressing the epsilon inhibitory fragment. Further, PKC delta, but not PKC epsilon, was specifically inhibited in the cells expressing the delta inhibitory fragment. Thus demonstrating that the delta and epsilon inhibitory fragments selectively inhibit PMA-induced translocation of their corresponding PKC isozymes.

Hundle et al [32] has shown, using these same cells, that chronic ethanol exposure enhances NGF-induced neurite outgrowth in control cells and cells expressing a dominant-negative inhibitor of PKC delta (DND cells) but not in cells expressing the inhibitor of the epsilon isoform of PKC (DNE cells). While it was not a goal of the current study to measure neurite outgrowth, daily observation of the cells was that neurite outgrowth was most dramatic in wild-type cells, slightly less so in the DND cells, and very limited in DNE cells (C. Reiter-Funk, personal observation). The work of Hundle et al [32] suggests that PKC epsilon but not PKC delta is involved in the effect of ethanol on neurite outgrowth. Therefore, we would have predicted that ethanol would cause an increase in microtubule content in DND cells but not in the DNE cells which do not have increased neurite outgrowth. However, we found that chronic ethanol enhances polymerization of tubulin into microtubules in dominant-negative PKC epsilon cells but not dominant-negative delta cells. Thus it appears that the delta isoform is involved in ethanol's enhancement of microtubule polymerization. Further, our data suggest that neurite outgrowth does not correlate with enhanced microtubule polymerization in PC12 cells. Interestingly, PKC delta has previously been shown to be involved in NGF-induced neurite outgrowth in PC12 cells [25]. It should be noted however, that our experimental paradigm varied slightly from that of Hundle et al [32]. For example, we allowed our cells to differentiate for 4 days prior to beginning ethanol. Therefore, apparent discrepancies of our findings with those of Hundle et al [32] could be related to these differences.

Based on our data, we speculate that ethanol's enhancement of microtubule polymerization may involve phosphorylation of tubulin by PKC. However, it should be noted that we have, thus far, not directly measured phosphorylation of tubulin. Studies have shown that chronic ethanol increases expression of delta and epsilon PKC in PC12 cells [30] and PKC activation enhances tubulin polymerization into microtubules [16-19]. Alternatively, chronic ethanol could be acting to alter important microtubule associated proteins. Many of these proteins, including Microtubule-associated Proteins (MAPS) are important for promoting microtubule assembly [34,35] and it has been shown that PKC phosphorylation mediates the assembly-promoting activity of these proteins [21,22,36,37]. Further studies are required to determine the mechanism of ethanol's enhancement of microtubule formation and the apparent role of delta PKC.

## Conclusion

Our studies demonstrate that chronic ethanol alters the relative ratio of free versus microtubule-associated tubulin content in PC12 cells, resulting in an increase in microtubule content and a corresponding decrease in free tubulin. This alteration was found to occur in wild type cells, as well as those expressing a dominant-negative inhibitor of epsilon PKC but not in cells expressing a dominant-negative inhibitor of delta PKC. These ethanol-induced changes could be important during activity-dependent remodeling of synapses or developmental growth of axons and dendrites which may lead to cognitive dysfunction.

## Methods

### Materials

Wild type PC12 cells were a gift from Nicholas Pantazis, Ph.D (University of Iowa). PC12 cell lines expressing dominant negative inhibitors of delta or epsilon PKC were a gift from Robert Messing, M.D (University of California, San Francisco). RPMI, Dulbecco's Modified Eagle Medium, and PenStrep were purchased from Gibco, NGF from R&D Systems, horse and fetal bovine serum was purchased from Hyclone, and laminin was purchased from Invitrogen. The microtubule assay kit was purchased from Cytoskeleton, Inc (#BK038).

### Cell Culture

PC12 cells were cultured in RPMI medium supplemented with 10% horse serum, 5% fetal bovine serum, and 1% PenStrep. Medium for the dominant-negative PC12 cells also included G418 (250 μg/ml) for selection purposes. The dominant-negative lines are cells that have been transfected to express isozyme specific dominant-negative inhibitors of delta or epsilon PKC and have been used to show specific PKC isozyme involvement in multiple ethanol effects [32,33]. These cell lines stably express the fragments δV1 or εV1, which are derived from the first variable domains of delta PKC or epsilon PKC, respectively. There was also a cell line transfected with vector alone, which served as a control. Dr. Messing has shown that these fragments can function as isozyme specific inhibitors [32,33].

The cells were maintained in an incubator at 37°C in 5% CO_2_. For all experiments, PC12 cells were plated into six-well, laminin-coated (5 μg/ml) plates at a density of 270,000 cells/well. The cells were differentiated into neuronal-like cells with Nerve Growth Factor (25 ng/ml) for four days prior to addition of any chronic drug exposure. For chronic exposures, ethanol (100 mM) was added to the culture media (along with NGF) for 96 hours and plates (control and ethanol) were wrapped in parafilm to prevent ethanol evaporation. Media were changed every other day.

### Microtubule assay

Measurement of microtubule and free-tubulin contents in cells are well-established assays. The materials are available as a commercially kit (Cytoskeleton, Inc). Microtubules are very sensitive to changes in temperature; therefore, all equipment and buffers were warmed to 37°C before use (unless otherwise indicated). To prevent free tubulin from polymerizing onto existing microtubules during the assay, lysis buffer was added at a ratio of 10 volumes of buffer to 1 volume of cell pellet. Cells were homogenized via syringe trituration and incubated for 10 minutes in lysis buffer (contents listed below). 10 μL of cell homogenates were saved for protein measurement using the Bradford Assay. Homogenized cells were then centrifuged at 100,000 × g for 30 minutes to separate microtubules from free-tubulin. The polymerized microtubules settle in the pellet, while the free-tubulin remains in the supernatant. Following centrifugation, the supernatant (free-tubulin) was removed and frozen until Western blot analysis. The pellet was resuspended in ice-cold water containing CaCl_2 _(200 μM) and incubated for one hour. CaCl_2 _acts to enhance microtubule depolymerization. Thus, the microtubules remaining in the pellet were depolymerized to free-tubulin. The samples were then centrifuged at 14,000 × g (4°C) for 10 minutes. The supernatant (containing free-tubulin representing the original microtubules) was collected and frozen. Tubulin concentrations in both fractions were measured using Western blotting as described below.

### Buffer contents

Lysis buffer: LMS1 solution containing GTP (100 mM) + ATP (100 mM) + protease inhibitor cocktail (10 μM) + Okadaic Acid (100 nM).

LMS1: PIPES (100 mM) containing MgCl_2 _(5 mM) + EGTA (1 mM) + glycerol (30%)+ Nonidet P40 (0.1%) + Triton X-100 (0.1%) + Tween 20 (0.1%) + beta-mercapto-ethanol (0.1%) + Antifoam (0.001%).

Protease inhibitor cocktail: Pepstatin (1 μg/ml) + Leupeptin (1 μg/ml) + benzamidine (10 μg/ml) + tosyl arginine methyl ester (500 μg/ml).

### Measurement of tubulin concentrations

Western blot analysis was used to determine tubulin content. Samples for Western blot were boiled with electrophoresis sample buffer (containing 25% glycerol, 5% beta-mercaptoethanol, 2% SDS, and 0.01% bromophenol blue in 62.5 mM Tris-HCl, pH 6.8). Equal amounts of protein (25 μg) were separated by SDS-PAGE using a 12% polyacrylamide gel. Molecular weight standards were also loaded. The separated proteins were transferred to nitrocellulose membranes using an electroblotting apparatus. The membrane was blocked [Tris-Buffered Saline (TBS) containing 4% nonfat dry milk and 0.05% Tween-20] for 20 minutes at room temperature. Membranes were then incubated in primary antibody (500 ng/ml; monoclonal anti-beta Tubulin; Cytoskeleton, Inc) overnight at 4°C. Following incubation in the primary antibody, membranes were washed with TBS/0.05% Tween-20 and incubated in secondary antibody (1:1000 dilution; HRP conjugated anti-mouse antibody; Santa Cruz). Membranes were again washed and tubulin bands were visualized by Enhanced Chemiluminescence (ECL) using standard luminol reagents captured using a BioRad Gel Doc 1000. The computer analysis software Molecular Analyst (version 2.1; BioRad) was used to quantify tubulin concentrations (relative density using the Volume Analysis setting with equal sized rectangular boxes set around individual bands). All blots were very clean and no filtering was needed.

### Data expression and statistical analysis

Tubulin concentrations in both the free-tubulin and microtubule fractions were expressed as a percent control relative density and presented as mean (+/-SEM). The data represent multiple replicates of the same experiment (n values listed in figure legends). Student's t-tests were used to make comparisons between control and ethanol-treated PC12 cells.

Each individual band analyzed was an independent extract from the same experiment, with the experiment being replicated 5 times. Each replication of the experiment was composed of 2 separate wells of cells for each treatment condition. For each of the 5 replications, the 2 control bands were averaged and each sample, including the controls, from that replication normalized to the average. The data was combined after all 5 replications.

## Authors' contributions

CKR-F conceived and performed the experiments and participated in writing the manuscript. DPD participated in the design of the study and writing of the manuscript. Both authors read and approved the final manuscript.
